# Feasibility, effectiveness, and safety of simultaneous side-by-side deployment of uncovered self-expandable metal stents for malignant hilar biliary obstruction: a retrospective single-center study

**DOI:** 10.1186/s12876-025-04053-0

**Published:** 2025-06-04

**Authors:** Chengcheng Christine Zhang, Marcus Kantowski, Cyrill Wehling, Patrick Michl, Ronald Koschny, Peter Sauer

**Affiliations:** 1 Department of Gastroenterology, University Hospital Heidelberg, Im Neuenheimer Feld 410, 69120 Heidelberg, Germany; 2 Department of General, Visceral and Transplantation Surgery, University Hospital Heidelberg, Im Neuenheimer Feld 420, 69120 Heidelberg, Germany

**Keywords:** Malignant hilar biliary obstruction, Percutaneous Endoscopic rendezvous technique, Recurrent biliary obstruction, Side By Side placement, Uncovered self, Expandable metal stent

## Abstract

**Background:**

Malignant hilar biliary obstruction is associated with a poor prognosis, making biliary drainage important for improving the quality of life. Endoscopic simultaneous side-by-side deployment of uncovered self-expandable metal stents is a novel approach. However, reliable clinical data on this method are limited. This retrospective, single-center study aimed to evaluate the feasibility, effectiveness, and safety of simultaneous side-by-side deployment of self-expandable metal stents for malignant hilar biliary obstruction.

**Methods:**

Data from all patients treated for malignant hilar biliary obstruction at our institution between May 2019 and February 2023 were retrospectively analyzed. The primary endpoints were the technical and clinical success rates, while the secondary endpoints included complications, recurrent biliary obstruction, time to recurrent obstruction, reintervention rate, and mortality.

**Results:**

Fifteen patients (mean age, 63 years; 8 men) were treated with simultaneous side-by-side deployment of metal stents for malignant hilar biliary obstruction. The location of the biliary obstruction was classified as Bismuth type III (*n* = 1) or IV (*n* = 14). Technical and clinical success rates were both 100%. Three patients underwent side-by-side placement of uncovered self-expandable metal stents using the combined percutaneous endoscopic rendezvous technique. The complication rate was 13.3%, with two patients experiencing recurrent biliary obstruction and cholangitis. The median time to recurrent obstruction was 97.5 days (range: 93–102 days). Both patients required reintervention. Moreover, the 30-day mortality rate was 6.7% (*n* = 1).

**Conclusions:**

Endoscopic simultaneous side-by-side deployment of uncovered self-expandable metal stents for unresectable malignant hilar biliary obstruction is feasible and safe, with high success rates. This technique not only effectively controls symptoms through successful biliary drainage, but can also be a promising option for complex anatomic situations when combined with the percutaneous endoscopic rendezvous technique.

## Background

Malignant hilar biliary obstruction (MHBO) is associated with a poor prognosis, making biliary drainage important for controlling cholangitis and improving patients’ quality of life.

To date, different plastic stents or self-expanding metal stents (SEMS) have been used for the endoscopic drainage of unresectable malignant biliary obstructions. However, multiple studies and meta-analyses indicate that SEMSs provide longer stent patency, lower complication and occlusion rates, lower reintervention rates, and costs compared with plastic stents in patients with distal or proximal malignant biliary obstruction [1–7]. Therefore, SEMS placement is preferred for malignant biliary obstructions. Another important issue regarding MHBO is determining the appropriate drainage area, which remains controversial, although bilateral drainage is frequently required owing to the extent of biliary obstruction and hepatic function.

Furthermore, the technical procedure for the endoscopic placement of a biliary SEMS for MHBO is challenging, with no consensus on the optimal technique. Available options include the unilateral or bilateral placement of SEMS [6, 8, 9], with further variants for bilateral drainage by stent-in-stent (SIS) or side-by-side placement, either with simultaneous or sequential deployment [6, 10–16]. The optimal placement method for MHBO has been debatable, with both side-by-side and stent-in-stent placements presenting technical challenges. The main difficulty lies in the insertion of the second SEMS. If it cannot be positioned correctly, the intended biliary drainage area cannot be reached, resulting in inadequate biliary drainage. Adjusting SEMS placement in these cases is difficult, as correcting or replacing the first SEMS is challenging.

Endoscopic simultaneous side-by-side deployment of SEMS is a novel concept [15, 16]. The advantage of this method is the simultaneous deployment of the two SEMSs, thereby avoiding the risk of dislocation of the first SEMS and misplacement of the second if placed sequentially. However, reliable clinical data regarding this approach are limited. Therefore, this retrospective, single-center study aimed to evaluate the feasibility, effectiveness, and safety of simultaneous side-by-side deployment of SEMS for unresectable MHBO.

## Methods

### Study design and population

In this single-center study, data from all patients treated between May 2019 and February 2023 were retrospectively analyzed. Data acquisition and evaluation were approved by the local ethics committee of Heidelberg University (S-043/2011) and conformed to the ethical guidelines of the Declaration of Helsinki. Written informed consent for endoscopic intervention was obtained from each patient or their legally authorized representatives.

The location of hilar biliary obstruction was classified according to the Bismuth classification [17]. The inclusion criterion was patients with unresectable MHBO classified as Bismuth III or Bismuth IV. Patients with an atrophic lobe were excluded, as these patients usually underwent unilateral stenting of the non-atrophic lobe. Demographic and clinical patient characteristics were retrieved, and procedural data were collected and analyzed.

Before endoscopic simultaneous side-by-side SEMS deployment, patients underwent multidetector computed tomography or magnetic resonance cholangiopancreatography, along with endoscopic retrograde cholangiopancreatography (ERCP), to identify the location of the hilar biliary obstruction and to assess the anatomy (e.g. evaluation of lobe atrophy). Fourteen patients underwent temporary biliary drainage via the placement of plastic stents before simultaneous side-by-side SEMS placement to assess the functional impact of hilar biliary drainage. In cases where ERCP was not possible beforehand because of specific reasons (such as biliary cannulation failure or duodenal stenosis), patients underwent percutaneous transhepatic biliary drainage (PTBD) followed by an endoscopic rendezvous procedure.

The follow-up period was retrospectively tracked from side-by-side SEMS placement until death or the date of the last follow-up.

### Primary and secondary endpoints

The primary endpoints of the study were the technical and clinical success rates. The secondary endpoints included the complication rate, recurrent biliary obstruction rate, time to recurrent biliary obstruction, reintervention rate, 30-day mortality, and overall mortality.

Technical success was defined as a successful endoscopic simultaneous side-by-side SEMS deployment at the intended biliary location across the papilla. Clinical success was defined as clinical improvement of the patients and ≥ 50% decrease in or normalization of the bilirubin level within 7 days of endoscopic side-by-side SEMS placement compared with bilirubin levels prior to biliary drainage.

Complications included obstruction, cholangitis, pancreatitis, and bleeding. Recurrent biliary obstruction was defined as stent occlusion due to tumor ingrowth, sludge, food impaction, and other factors. The time to recurrent biliary obstruction was categorized as early (≤ 30 days) or late (≥ 31 days) from endoscopic side-by-side SEMS placement to the onset of recurrent biliary obstruction [18].

### Simultaneous side-by-side deployment using SEMSs

**Fig. 1:**
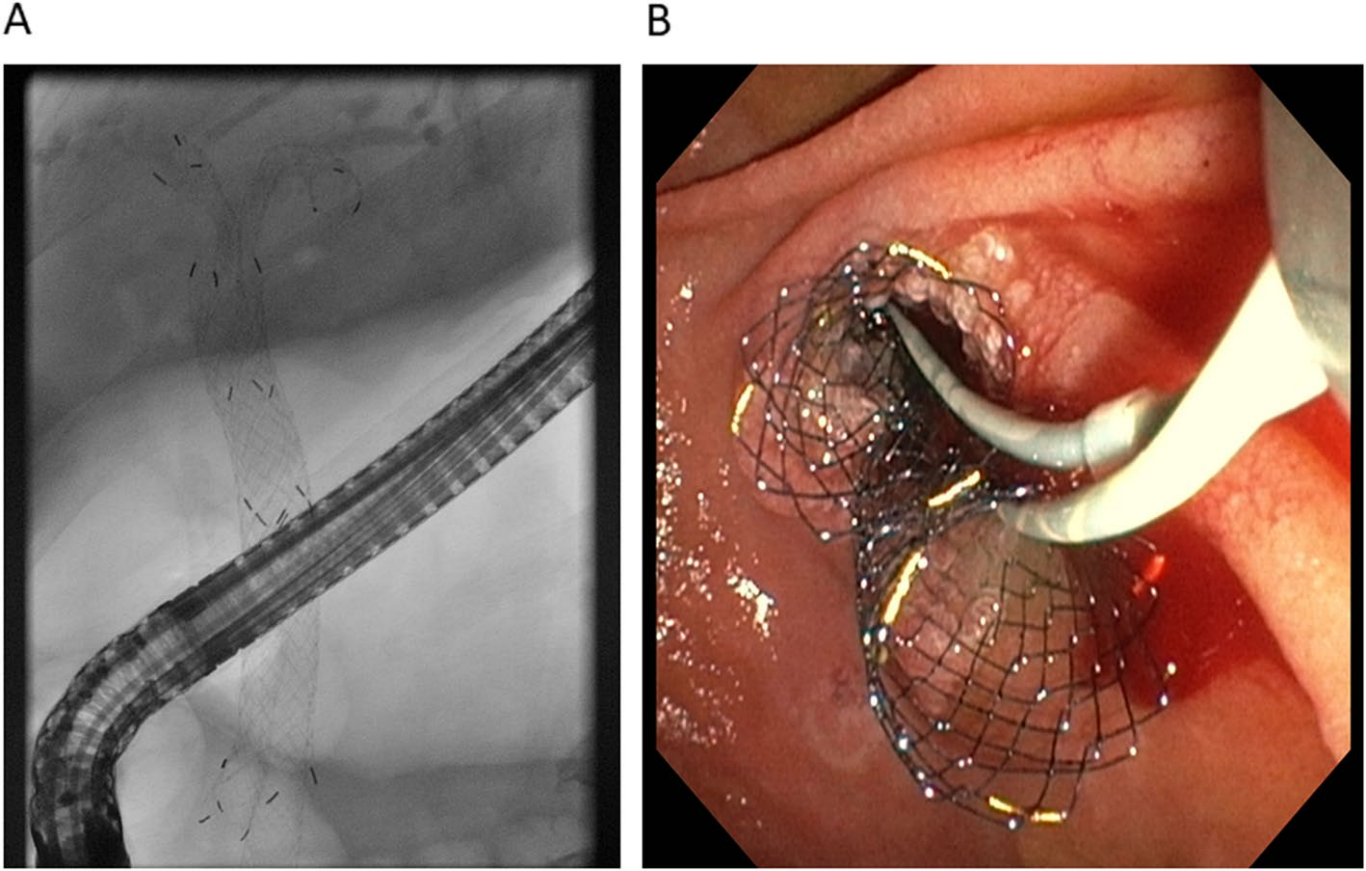
Endoscopic simultaneous deployment of biliary side-by-side SEMSs: (**a**) cholangiogram and (**b**) endoscopic image. The SEMSs were released simultaneously from the left and right intrahepatic bile ducts across the duodenal papilla. SEMS: self-expandable metal stent

All patients underwent endoscopic bile duct cannulation during ERCP using two 0.025-inch guidewires inserted into the left and right intrahepatic bile ducts through the MHBO. Patients with a very narrow biliary obstruction underwent biliary dilation using a 12- or 18-Fr biliary balloon dilation catheter. Each SEMS was separately placed in the left and right intrahepatic bile ducts. For successful simultaneous deployment of the SEMSs an exact coordination of the endoscopist and two assistants are crucial. Each SEMS was then released by one assistant under both x-ray guidance und endoscopic control instructed by the endoscopist. Finally, both SEMSs were released simultaneously across the duodenal papilla (Fig. 1). The length of the stent placed (100 or 120 mm) was estimated based on the distance between the proximal end of the hilar biliary obstruction and the duodenal papilla on cholangiography. Experienced endoscopists and assistants performed all ERCP procedures.

### Devices

We used SEMSs with an 8-mm diameter, 100- or 120-mm length, and a 5.9-Fr delivery device (Hanarostent ® biliary stent; Medical Systems Corp., Tokyo, Japan). ERCP was performed using a duodenoscope (TJF-Q180 V or TJF-Q190 V; Olympus Medical Systems Corp., Tokyo, Japan). All patients underwent ERCP procedures under deep sedation with propofol and remifentanil. Furthermore, an ERCP catheter (Contour™ ERCP Cannula; Boston Scientific, Natick, MA, USA) was used for bile duct cannulation, while 0.025-inch Guidewires (VisiGlide 2; Olympus Medical Systems Corp.) were used for selective bile duct cannulation and intrahepatic bile duct insertion. A 12- or 18-Fr biliary balloon dilation catheter (Olympus Medical Systems Corp.) was used for biliary dilation.

### Percutaneous endoscopic rendezvous technique

If access to the intrahepatic bile ducts was not achievable owing to biliary cannulation failure or duodenal stenosis, patients underwent percutaneous endoscopic rendezvous procedures. In such cases, patients first underwent percutaneous transhepatic biliary drainage, followed by ERCP with internalization of the biliary drainage through endoscopic placement of side-by-side SEMSs via the rendezvous technique.

### Reintervention and management for recurrent biliary obstruction

Reintervention was performed if stent obstruction or cholangitis occurred after endoscopic side-by-side SEMS placement. Patients experiencing stent obstruction due to bile duct sludge, stones, or food impaction underwent endoscopic removal and extraction using a balloon catheter. If the stent obstruction was caused by tumor ingrowth, endoscopic plastic stent placement or stent-in-stent placement using SEMSs was performed.

### Statistical analyses

Descriptive statistics were calculated for all parameters. Results were expressed as means ± standard deviation or as median (interquartile range) for continuous variables. Categorical variables are presented as counts and percentages. Survival was estimated using the Kaplan–Meier method. The analyses were performed using SPSS software (SPSS Inc., Chicago, IL, USA, version 29.0).

## Results

**Fig. 2:**
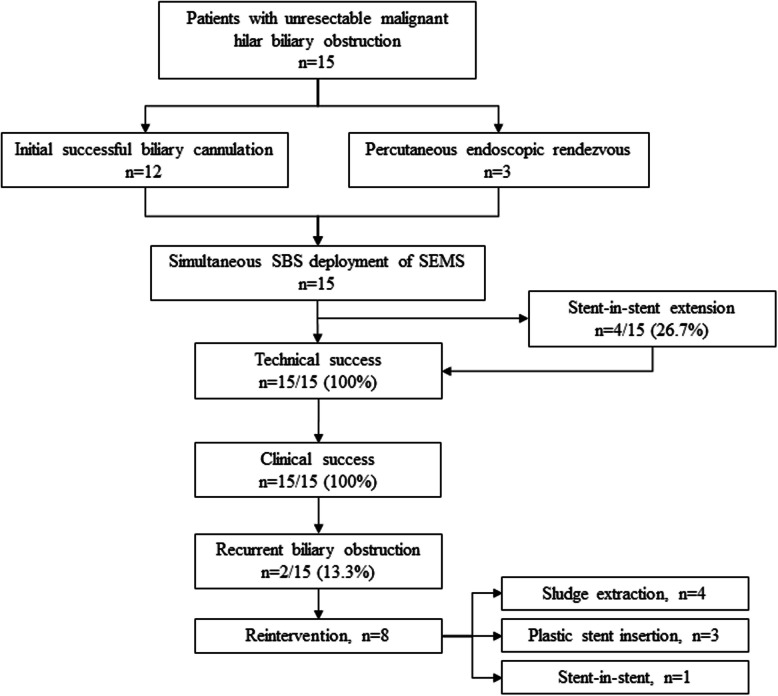
Flow diagram of the study. SBS: side-by-side; SEMS: self-expandable metal stent

Fifteen patients (eight men and seven women) with a mean age of 63 years underwent simultaneous side-by-side SEMS placement for unresectable MHBO at our institution between May 2019 and February 2023 (Fig. 2). Patient characteristics are shown in Table 1.

**Table 1:** Baseline characteristics

Number of patients, n	15
Mean age ± SD, years	63 ± 12
Male, n (%)	8 (53.3)
Etiology of MHBO, n (%)	
Cholangiocarcinoma	9 (60.0)
Metastatic Cancer	6 (40.0)
Bismuth classification, n (%)	
Type III	1 (6.7)
Type IV	14 (93.3)
Median initial serum bilirubin, mg/dl (range)	6.9 (1.1–22.9)
Median follow-up period, days [95% CI]	322 [100–544]

*SD* Standard deviation, *MHBO* Malignant hilar biliary obstruction, *CI* Confidence interval.

The etiology of MHBO was cholangiocarcinoma in nine patients (60%) and metastatic cancer in six patients (40%). The locations of MHBO were classified as Bismuth III (*n* = 1; 6.7%) or Bismuth IV (*n* = 14; 93.3%). The Bismuth classification was applied to MHBO of metastatic cancer in analogy to cholangiocarcinoma. The median initial serum bilirubin level before any intervention was 6.9 mg/dL (range: 1.1–22.9 mg/dL).

### Feasibility: Technical success

As shown in Table 2, the technical success rate was 100% (*n* = 15/15).

**Table 2:** Feasibility and effectiveness of simultaneous side-by-side SEMS placement. Technical and clinical success data are shown. SEMS: self-expandable metal stent

Technical success, n (%)	15 (100)
Clinical success, n (%)	15 (100)
Dilatation prior stenting, n (%)	2 (13.3)
Rendezvous technique, n (%)	3 (20.0)
Length of the SEMS, right/left, n (%)	
100 mm/100 mm	6 (40.0)
120 mm/120 mm	7 (46.7)
120 mm/100 mm	0 (0)
100 mm/120 mm	2 (13.3)
Extension by stent-in-stent placement, n (%)	4 (26.7%)
Median procedure duration, min (range)	45 (30–104)

Two patients (*n* = 2/15, 13.3%) underwent biliary balloon dilation before side-by-side placement, while three patients (*n* = 3/15, 20.0%) underwent a percutaneous endoscopic rendezvous procedure because of extremely narrow bile ducts and failure of biliary cannulation. One of these three patients also showed significant duodenal stenosis due to advanced pancreatic carcinoma and had previously undergone duodenal stenting. Additionally, four patients underwent stent-in-stent placement for extension because the initial endoscopic simultaneous side-by-side SEMS placement did not reach the intended biliary location across the papilla, primarily due to misjudgment of the biliary length (*n* = 4/15, 26.7%). Detailed data on the lengths of the selected SEMS are shown in Table 2. Successful final SEMS placement across the papilla, after potential correction, was achieved in all patients (*n* = 15/15, 100%). The median procedure duration was 45 min (range: 30–104 min).

### Effectiveness: Clinical success and course of bilirubin levels

**Fig. 3:**
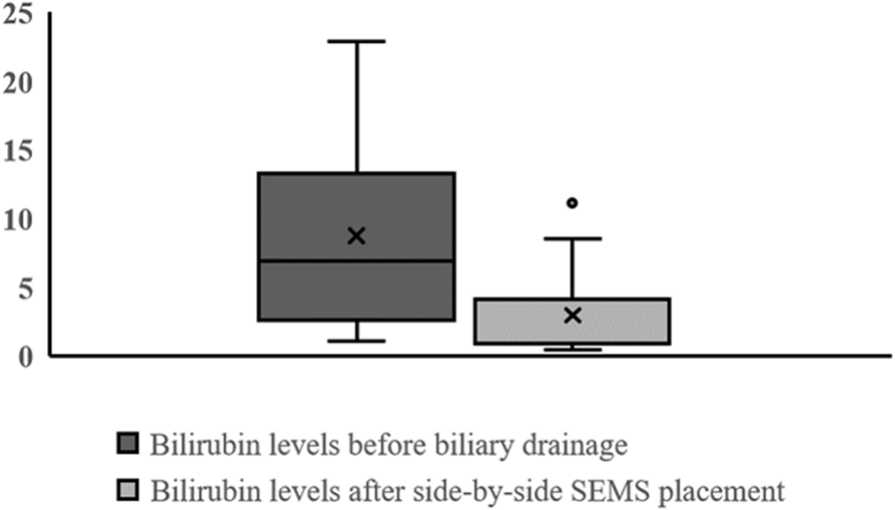
Bilirubin levels of patients. Bilirubin levels before biliary drainage and within 7 days of simultaneous side-by-side SEMS placement. SEMS: self-expandable metal stent

As shown in Table 2, the clinical success rate was 100% (*n* = 15/15). All patients showed clinical improvement and at least a 50% decrease in or normalization of the bilirubin level within 7 days of endoscopic side-by-side SEMS placement compared with bilirubin levels prior to biliary drainage. Figure 3 illustrates the median and mean initial bilirubin levels of the patients before endoscopic intervention and the median and mean bilirubin levels 7 days after simultaneous side-by-side SEMS placement.

### Safety: Complications, recurrent biliary obstruction, and reinterventions

Two patients (*n* = 2/15, 13.3%) developed complications after side-by-side SEMS placement (Table 3).

**Table 3:** Safety and outcomes of simultaneous side-by-side SEMS placement

Patients with complications, n (%)	2 (13.3)
Overall complications, n	4
Obstruction	2
Cholangitis	2
Early complications (≤ 30 days)	0
Late complications (≥ 30 days)	4
Median time to recurrent obstruction, days (range)	97.5 (93–102)
Dysfunction-free patency duration, mean [95% CI], days	361 [255–466]
Patients with reintervention, n (%)	2 (13.3)
Reintervention, n	8
Biliary stone/sludge extraction, n	4
Plastic stent placement, n	3
Stent-in-stent placement, n	1
Follow-up period, median [95% CI], days	322 [100–544]
30-day mortality, n (%)	1 (6.7)
Overall mortality, n (%)	7 (46.7)

*CI* Confidence interval.

**Fig. 4:**
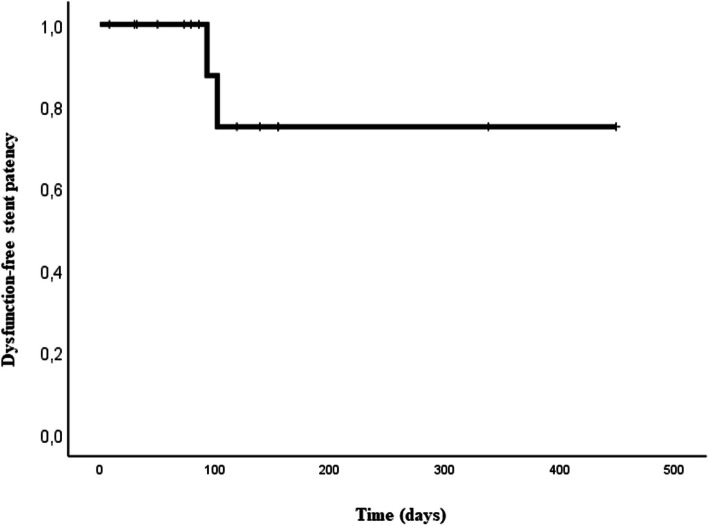
Kaplan–Meier graph showing the dysfunction-free stent patency of patients who underwent simultaneous deployment of biliary side-by-side self-expandable metal stents

Both patients experienced recurrent biliary obstruction accompanied by cholangitis after a median of 97.5 days (range: 93–102 days). The time to recurrent biliary obstruction from endoscopic side-by-side SEMS placement to the onset of recurrent biliary obstruction was considered late (≥ 31 days) in both patients. Kaplan–Meier analysis revealed that the mean dysfunction-free patency duration was 361 days (95% CI: 255–466) in all patients (Fig. 4).

The abovementioned patients underwent endoscopic reintervention after simultaneous side-by-side SEMS placement. They underwent sludge or stone extraction and removal of food impaction using an endoscopic balloon catheter. Additionally, cholangitis was treated with antibiotics. One patient showed sustained occlusion-free success without further complications after one reintervention for 229 days until death. However, the other patient required seven reinterventions due to recurrent biliary obstruction, necessitating repeated extraction of sludge or stones and impacted food. Eventually, additional stent-in-stent placement of SEMS was performed owing to tumor disease progression with tumor ingrowth and consecutive stent occlusion. Since then, the patient has shown sustained occlusion-free success without further complications after the last reintervention for 173 days until the last follow-up.

No other complications besides the abovementioned recurrent biliary obstruction and cholangitis were observed in our study cohort.

The median follow-up period was 322 days (95% CI: 100–544) after the procedure. The 30-day mortality was 6.7% (*n* = 1/15), and overall mortality was 46.7% (*n* = 7/15). Cancer progression was the major cause of death in all patients.

## Discussion

This study demonstrated the feasibility, effectiveness, and safety of endoscopic side-by-side SEMS placement for unresectable MHBO.

Different studies and meta-analyses have revealed the superiority of SEMS placement over plastic stent placement for biliary drainage in patients with distal or proximal malignant biliary obstruction in terms of recurrent occlusion and reintervention rates [1–6]. Additionally, the question of adequate stent quality and the optimal biliary drainage area when comparing unilateral and bilateral stenting remains controversial in MHBO. Different studies have addressed this topic, demonstrating a higher technical success rate with unilateral stenting but better outcomes of cumulative stent patency with bilateral stenting [9, 19]. In this context, accurate positioning of SEMSs for MHBO is essential to ensure optimal biliary drainage of the liver. However, the optimal technique for this technically challenging endoscopic procedure remains controversial.

Various techniques have been described for bilateral stenting (Table 4). One method is the stent-in-stent placement of a second SEMS into the contralateral intrahepatic bile duct through the meshes of the first SEMS. This method is challenging, as the wires of the mesh must be adequately dilatated for the insertion of the second stent. Moreover, reported technical success rates for expert endoscopists range from 80–100% [20–23]. Another development is the Y-stent for hilar placement, a 10-mm diameter SEMS with a median patency period of 217 days (range: 55–365). However, this development is associated with only an 80% technical success rate and difficulties for later stent revisions in cases of tumor ingrowth and biliary obstruction [24, 25].

**Table 4:** Studies of bilateral hilar drainage

Author	Patients (n)	Endoscopic technique	Technical success rate % (n)	Clinical success rate % (n)	Stent occlusion rate % (n)	Stent patency (months)	Follow-up (months)
Chahal et al. [21]	21	SIS	100 (21/21)	100 (21/21)	38 (8/21)	6.3	6.1
Park et al. [22]	35	SIS	94 (33/35)	100 (33/33)	6 (2/33)	5	4.7
Lee et al. [24]	10	SIS	80 (8/10)	100 (8/8)	25 (2/8)	7.2	7.1
Kim et al. [25]	34	SIS	85 (29/34)	100 (29/29)	31 (9/29)	6.2	7.9
Lee et al. [26]	84	SIS	95 (80/84)	93 (78/84)	31 (24/78)	7.9	8.4
Kogure et al. [27]	12	SIS	100 (12/12)	92 (11/12)	50 (6/12)	6.7	5
Hwang et al. [28]	30	SIS	87 (26/30)	87 (26/30)	39 (10/26)	4.7	5.8
Dumas et al. [29]	45	Sequential SBS	73 (33/45)	100 (33/33)	3 (1/33)	N/A	7.8
Lee et al. [30]	44	Sequential SBS	91 (40/44)	98 (39/40)	45 (18/40)	5.2	5.9
Cheng et al. [31]	36	Sequential SBS	97 (35/36)	N/A	31 (11/35)	5.6	4.8
Inoue et al. [13]	17	Simultaneous SBS	100 (17/17)	100 (17/17)	47 (8/17)	4.7	N/A
Chennat et al. [14]	10	Simultaneous SBS	100 (10/10)	N/A	25 (4/10)	4.3	7
Law et al. [15]	24	Simultaneous SBS	71 (17/24)	N/A	50 (12/24)	2.9	3.2
Kawakubo et al. [16]	13	Simultaneous SBS	85 (11/13)	N/A	38 (5/13)	8.7	6.4

*SIS*: Stent-in-stent, *SBS* Side-by-side, *N/A* Not available.

Currently, the most common technique is the side-by-side placement of SEMS. Different studies described the outcomes of sequential SEMS deployment with side-by-side placement for MHBO, with technical success rates ranging from 50–97% [29, 31]. The main challenge with this technique is the adequate deployment of the second SEMS in the correct position without entanglement of the two separate guidewires or dislocation of the primarily inserted SEMS. Furthermore, another difficulty of the sequential deployment might be the positioning of the second device, as the already released first SEMS could represent an obstacle to the passage. All these factors can result in inadequate biliary drainage and offer limited possibilities for correcting SEMS locations. Recent innovations in endoscopic technology have been developed to facilitate the side-by-side placement of SEMSs. Endoscopic simultaneous side-by-side deployment of SEMS represents a novel concept [6, 13–16]. The significant advantage of this technique is the theoretical prevention of problems when inserting the second stent, as the two SEMSs are released simultaneously. The simultaneous deployment of side-by-side stents has become more feasible with the development of different ≤ 6-Fr delivery devices. Some studies reported shorter procedure time and higher technical success rate than sequential deployment [6, 13, 15]. However, comprehensive clinical data supporting this technique remains limited.The findings from this study demonstrate that the simultaneous deployment of side-by-side SEMS placement using a 5.9-Fr delivery device for MHBO is technically feasible, safe, and effective, even for complex anatomic situations, when combined with the percutaneous endoscopic rendezvous technique. The technical success rate in our study was 100% (*n* = 15/15). Alignment of both distal SEMS ends is essential to facilitate reintervention in cases of recurrent biliary obstruction or other complications [32]. Our study highlights the successful simultaneous side-by-side deployment of SEMS in three patients with initial failure of biliary cannulation due to extremely narrow bile ducts. These patients underwent successful simultaneous side-by-side SEMS placement using a percutaneous endoscopic rendezvous procedure. One of these three patients also showed significant duodenal stenosis and had previously undergone duodenal stenting. These results indicate that simultaneous side-by-side SEMS deployment can be successfully performed in patients with complex anatomic situations.

The findings of our study regarding success rates are consistent with previously reported technical success rates of different studies, ranging from 71–100% [13–16]. Law et al. reported successful deployment of both stents across the identified hilar stricture in 70.8% (*n* = 17/24). However, simultaneous deployment, which resulted in the alignment of the distal stent markers, was achieved in only 53% of the procedures [15]. The authors used the Zilver® SEMS system (Cook Japan, Tokyo, Japan) with stent lengths of 80, 60, and 40 mm. In a study by Kawakubo et al., the technical success rate (defined as single-step simultaneous side-by-side double SEMSs placement across the stricture) of 84.6% was achieved (n = 11/13) [16]. However, initial placement across the papilla could not be achieved in any patient, and the authors did not describe any further adjustments to extend the distal ends across the papilla. The authors used the 6-Fr delivery system measuring 10 mm in diameter and 80 mm in length from Zilver®. Chennat et al. reported an overall technical success rate of 100% for simultaneous side-by-side SEMS placement in 10 patients [14]. However, the bilateral hilar stents could not bridge the ampullary region in all patients, and all 10 patients required additional distal stent placement. The authors also used the Zilver® SEMS system but did not provide a detailed description of the diameters and lengths of stents that they used. Inoue et al. reported an overall technical success rate of 100% for simultaneous side-by-side SEMS placement in 17 patients with MHBO [13]. The length of the SEMS was selected based on the length of the stenosis, with all SEMSs positioned above the duodenal papilla. Subsequently, alignment of the distal stent ends was attempted successfully in 16 out of 17 patients (94%). The study used 5.7-Fr SEMSs (BileRush Selective; Piolax Medical Devices, Kanagawa, Japan) with a diameter of 8 mm.

The Hanarostent® biliary stent system used in this study is currently available with SEMS of 6- or 8-mm diameter and stent lengths of 40, 60, 80, 100, and 120 mm. However, we used only SEMSs of 8-mm diameter and 100- or 120-mm length. Two cases without initial SEMS bridging across the papilla received 100-mm stents, while the other two patients received 120-mm stents, also without reaching the papilla. The first two cases might have been successful with initial SEMS placement across the papilla using 120-mm stents instead of 100-mm stents. However, owing to misjudgment of the real bile duct length, further stent extensions had to be performed. This misjudgment of the effective biliary length might be attributed to the pliability of the 5.9-Fr delivery device. Nevertheless, in the third and fourth patients, the 120-mm stents were not long enough to cover the stenosis and reach the papilla.

In this study, two patients experienced recurrent biliary obstruction after a median of 97.5 days (range: 93–102 days) due to sludge or food impaction and tumor ingrowth. Both patients underwent successful endoscopic reintervention after simultaneous side-by-side SEMS placement through either sludge extraction or stent-in-stent SEMS placement.

Kawakubo et al. noted stent dysfunction in five out of 13 patients (38.4%), attributed to tumor ingrowth (*n* = 4) and biliary sludge (*n* = 1) [16]. Endoscopic reintervention was successful in all five patients, with a median dysfunction-free patency duration of 263 days (95% CI: 12–263).

In a study by Chennat et al., 25% of the patients (*n* = 4/10) experienced recurrent biliary obstruction due to sludge formation (*n* = 1, early complication) and tumor ingrowth (*n* = 3, late complication) [14]. Three patients with tumor ingrowth required subsequent PTBD because endoscopic drainage was not feasible. Additionally, the patient with sludge received successful endoscopic reintervention through bilateral plastic stent insertions within the SEMS. The median duration of stent patency was 130 days (range: 64–168 days).

In Law et al.’s study, reinterventions due to tumor ingrowth (*n* = 10) and biliary sludge (*n* = 2) were required in 50% of the patients (*n* = 12/24) at a median of 98 days (range: 60–137 days) [15]. Successful endoscopic reintervention was achieved in nine out of 11 patients (81.8%), while three patients required PTBD. The overall stent patency duration was 86 days (range: 42–137 days).

Inoue et al. reported recurrent biliary obstruction in eight of 17 patients (47%) due to tumor ingrowth (*n* = 7) and sludge (*n* = 1) after a median time of 140 days [13]. Successful endoscopic reintervention was achieved in 86% (*n* = 6/7).

In our study, a stent occlusion rate of 13.3% was observed, which is lower compared to previously reported stent occlusion rates ranging from 18–53% [3, 11, 14, 15, 21, 33, 34]. This difference could be attributed to the placement of the SEMS across the papilla, which was conducted in all patients. This approach not only facilitates endoscopic access for possible reinterventions, but also improves free biliary drainage. This study has a few limitations. The monocentric design of this study and the small study cohort might limit the external validity and the generalizability of this study’s findings. Furthermore, this study did not compare the simultaneous side-by-side deployment technique directly with other established strategies (e.g., unilateral stenting, sequential stent-in-stent placement), making it difficult to quantify the benefits over conventional approaches. Additionally, only one 5.9-Fr delivery system was used; therefore, the clinical utility of simultaneous side-by-side deployment with other delivery systems should be investigated and compared. Furthermore, as this study focuses on the evaluation of the simultaneous side-by-side deployment of SEMSs for MHBO, we neither considered other etiologies of hilar strictures nor other techniques for biliary drainage for comparison. Nevertheless, to our knowledge, this is the first study to report the outcomes of simultaneous side-by-side deployment of SEMSs for MHBO and in complex anatomic situations when combined with the percutaneous endoscopic rendezvous technique. Due to the limitations of this study including its retrospective design, along with relatively short and varied patient follow-up durations, further multicenter, prospective, randomized controlled trials should be conducted to validate the benefits and long-term outcomes of simultaneous side-by-side SEMS deployment and to compare this special technique to conventional techniques for different patient populations.

## Conclusion

Endoscopic simultaneous side-by-side deployment of uncovered SEMSs in patients with unresectable MHBO is a feasible and safe approach. It improves the quality of life through successful biliary drainage. Moreover, it appears to be a promising option for addressing complex anatomic situations, particularly when combined with the percutaneous endoscopic rendezvous technique.

## Data Availability

The data that support the findings of this study are available from the corresponding author upon reasonable request.

## Abbreviations

MHBO: Malignant hilar biliary obstruction
ERCP: Endoscopic retrograde cholangiopancreatography
PTBD: Percutaneous transhepatic biliary drainage
SEMS: Self-expanding metal stents
